# Study on Flexible sEMG Acquisition System and Its Application in Muscle Strength Evaluation and Hand Rehabilitation

**DOI:** 10.3390/mi13122047

**Published:** 2022-11-22

**Authors:** Chang Liu, Jiuqiang Li, Senhao Zhang, Hongbo Yang, Kai Guo

**Affiliations:** 1 College of Mechanical and Electrical Engineering, Changchun University of Science and Technology, Changchun 130022, China; 2 Suzhou Institute of Biomedical Engineering and Technology, Chinese Academy of Sciences, Suzhou 215163, China; 3 School of Biomedical Engineering (Suzhou), Division of Life Sciences and Medicine, University of Science and Technology of China, Hefei 230026, China

**Keywords:** wearable devices, flexible sEMG acquisition system, muscle strength, rehabilitation, single-lead sEMG

## Abstract

Wearable devices based on surface electromyography (sEMG) to detect muscle activity can be used to assess muscle strength with the development of hand rehabilitation applications. However, conventional acquisition devices are usually complicated to operate and poorly comfortable for more medical and scientific application scenarios. Here, we report a flexible sEMG acquisition system that combines a graphene-based flexible electrode with a signal acquisition flexible printed circuit (FPC) board. Our system utilizes a polydimethylsiloxane (PDMS) substrate combined with graphene transfer technology to develop a flexible sEMG sensor. The single-lead sEMG acquisition system was designed and the FPC board was fabricated considering the requirements of flexible bending and twisting. We demonstrate the above design approach and extend this flexible sEMG acquisition system to applications for assessing muscle strength and hand rehabilitation training using a long- and short-term memory network training model trained to predict muscle strength, with 98.81% accuracy in the test set. The device exhibited good flexion and comfort characteristics. In general, the ability to accurately and imperceptibly monitor surface electromyography (EMG) signals is critical for medical professionals and patients.

## 1. Introduction

Although existing conventional surface electromyographic signal (sEMG) monitoring devices are able to meet the needs of medical and scientific research in terms of accuracy, they are large, expensive, not portable to detect, and require professional operation. It is also very difficult to guarantee the stability of their muscle electrical signal data to meet the needs of detection, even under stretchable conditions [1]. Flexible stretchable electronics can transform electronic components and circuits from rigid and rigid substrates to more flexible and stretchable devices. It is an emerging research science with promising applications [2]. In recent years, the research development of stretchable electrodes has driven the emergence of wearable electronics, electronic skin, implantable medical electronics, soft robots, and new flexible human–machine interfaces [3,4,5,6]. Therefore, the combination of flexible stretchable technology with the ability to withstand various mechanical deformations and surface EMG signal monitoring devices has become a new development trend [7,8].

The sensing system of sEMG mainly consists of an sEMG sensor and an sEMG acquisition circuit [9,10,11,12]. The sEMG sensor contacts the skin and collects surface EMG signals from muscle movements. Currently, the electrodes used for surface EMG acquisition mainly include wet and dry electrodes. The most common one in the market is a conductive gel silver/silver chloride (Ag/AgCl) wet electrode, which has problems such as short duration of use, biological toxicity, and skin irritation [13]. Dry electrodes are mainly divided into metallic electrodes and flexible electrodes. In recent years, with the rise of flexible electronic technology, flexible skin electrodes for bioelectric monitoring have been rapidly developed. Since the elastic modulus of flexible electrodes is similar to that of human skin, it can move and deform with the skin, which makes flexible electrodes more comfortable. Due to its good mechanical deformation properties, it can also guarantee that the flexible sEMG sensor does not produce relative displacement with the skin under the influence of breathing and other movements, thus avoiding the interference of noise such as movement artifacts generated by displacement and jitter between the electrode and the skin, and making the acquisition process more stable [14]. Currently, most flexible electrodes are composed of nano-conductive fillers and flexible substrates. Among them, the conductive fillers include carbon-based conductive materials such as graphene or carbon nanotubes, and nanometal-based conductive substrates such as gold, silver, and copper. The main flexible substrates used for sEMG sensors are polydimethylsiloxane (PDMS) and polyurethane (PU) [15,16,17,18,19]. Liu et al. [20] investigated flexible skin sEMG sensors using polyimide (PI) substrates and Cu serpentine interconnects in the field of oral recognition. A flexible sensing-based sEMG signal acquisition system was completed. High accuracy was achieved for specific spoken words by using a skin-like sEMG sensor and a neural network to accomplish action pattern recognition. Chandra et al. [21] developed a flexible skin sEMG electrode for high-density surface EMG recording based on flexible stretchable electronics with PI substrate and serpentine gold wiring. The performance of the fabricated sensor was tested and compared. The prepared flexible sEMG sensor can record sEMG signals accurately without significant distortion due to the electrode shape and size. The amplitude of the recorded sEMG signal is significantly increased and the signal-to-noise ratio is also improved compared with the commercially available electrodes. Clearly, the use of flexible dry electrodes instead of conductive Ag/AgCl wet electrodes for coupling to human skin facilitates the expansion of the monitoring area used for myoelectric signal recognition and improves human comfort.

The sEMG acquisition circuit mainly consists of signal acquisition, filtering, amplification, and transmission. sEMG electrodes acquire weak electrical signals that are filtered and amplified and the resulting sEMG signals are processed and transmitted. Fialkoff et al. [22] proposed a simple muscle signal acquisition method to estimate grip force. By obtaining the relationship between EMG and force, the grip force of the hand can be estimated from the reconstructed EMG image with some accuracy. However, they used eight Myoware EMG SEN-13723 sensors to measure the EMG signals and selected the Raspberry Pi for control, leading to the large size and wiring of their sEMG acquisition device; apparently the whole system is not portable. Tan et al. [23] prepared an sEMG electrode by using polyethylene terephthalate (PET) film with ZnS: Cu particles and PDMS composite. The sEMG electrodes were used to try algorithms for myoelectric signal acquisition and muscle force assessment. However, their system was more complex. After the sensors were connected to the amplifier circuit, the Arduino was used for ADC conversion and data transfer. The whole system uses a larger rigid circuit board and a large number of data lines, thus losing the advantage of the flexibility of the sensor. Obviously, the comfort and portability of the hardware circuit for signal acquisition and processing should also be considered while satisfying the need for accurate and highly reliable data acquisition. In general, the ability to accurately and imperceptibly monitor surface EMG signals is critical for medical professionals and patients.

Muscle force is an important physiological parameter of the human body; this has important applications in medical diagnosis, motor evaluation, and human–computer interaction [24,25]. However, the direct and accurate measurement of muscle force is currently very difficult and complex, and the study of muscle force estimation methods is of great significance. Numerous studies in the literature have shown that sEMG has a high correlation with muscle activity [26,27,28] and that the signal has the advantages of easy acquisition and non-invasiveness, making it suitable for estimating muscle force [29,30,31]. Muscle force is the result of the complex superposition of many muscle groups and it is very difficult to estimate the force of each muscle. For this reason, the internal muscle is treated as a whole, the contribution of each muscle is not subdivided, and its force on the external environment is treated as muscle force (sEMG muscle force model [32,33,34,35]). Li et al. [36] fitted the sEMG muscle force curve by polynomial. Bai et al. [37] used an artificial neural network (ANN) to establish the relationship between quadriceps and flexor muscle force. Huang et al. [38] constructed the mapping between muscle activation degree and flexor muscle force by fitting a second-order polynomial. Liang et al. [39] established a manual grasping force estimation model based on wavelet packets and support vector machine (SVM). Jalaludin et al. [40] established the relationship between an EMG signal and thumb pressure by ANN. In conclusion, many results have been achieved in muscle force estimation. There are also common problems such as low accuracy and lack of convenience, which researchers have been trying to explore in order to achieve accurate, fast, and convenient muscle force estimation [41,42].

Currently, the research on sEMG sensors is mainly focused on motion recognition, including limb muscles and facial muscles. Since different limb movements generate different muscle potentials, the acquisition of limb EMG can be used for movement recognition, while the acquisition of facial muscles is generally used for lipographic interaction [43,44,45]. Limb motion recognition using sEMG sensors can generally be applied in the field of physical rehabilitation by recognizing movements and by controlling rehabilitation robots [46,47,48,49,50,51,52,53]. Xiao et al. [54] extracted three channels of EMG signals using root mean square eigenvalues and trained recognition algorithms for seven specific hand gestures. The high accuracy of gesture recognition was achieved by myoelectric signal feature extraction. They applied the EMG signals to the control of a hand-functional robot with an exoskeleton. By extracting the three-channel surface EMG signal and by extracting the eigenvalue algorithm, they achieved the average real-time motion intention recognition.

In general, although there are some studies on the application of sEMG acquisition systems, there are still some shortcomings. Some studies use commercially available sEMG development board modules, which are not well-adapted to the characteristics of the human arm. Most studies use wired connections and more wires are very limiting for application scenarios. Many researchers have paid little attention to the overall flexibility of muscle strength assessment systems, including the sensors for sEMG acquisition and the hardware for wireless sEMG acquisition modules. Refs. [55,56,57,58,59] combined the problems of existing surface EMG detection products. This paper designed a graphene-based flexible dry electrode as the acquisition electrode and designed and fabricated a surface EMG acquisition system device with flexible signal acquisition and processing hardware. The acquisition device has the characteristics of accurate data acquisition, high reliability, small size, low power consumption, simple operation, no sense of violation to wear, etc. It can do long-term monitoring of EMG signaling in daily life work and the signal is transmitted to the host computer in real time through Bluetooth. Finally, it is applied to muscle strength assessment and hand rehabilitation training in this paper.

## 2. Materials and Methods

In this section, the overall preparation process and several performance test experiments (mechanical properties and grip force test) of the flexible sEMG acquisition system will be presented. By using flexible electronics, flexible sEMG electrodes based on graphene are designed and combined with a flexible stretchable substrate to make sEMG sensors. Flexible sEMG wireless acquisition hardware is designed to collect sEMG signals under different grip forces or different hand gestures. The sEMG system is applied to grip force estimation and rehabilitation by designing algorithms and control programs.

### 2.1. Preparation of Flexible sEMG Sensors

**Figure 1:**
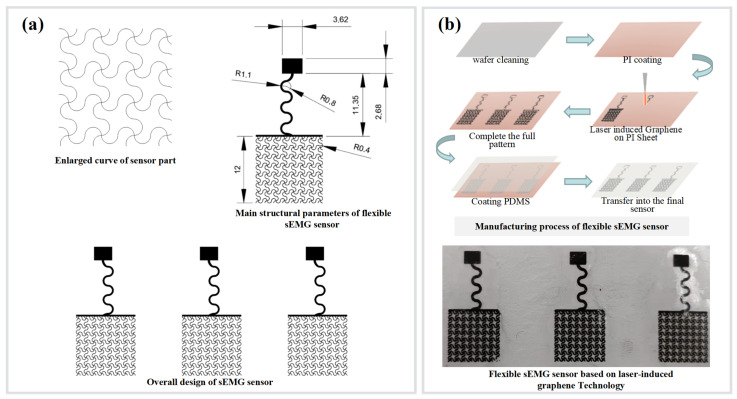
Process of flexible stretchable myoelectric sensor preparation. (**a**) The process of designing the lead transmission line part of the sensor using flexible serpentine and the sensor acquisition interface part. (**b**) The manufacturing process of a flexible sEMG sensor.

The flexible sEMG sensor consists of a square-shaped electrode with a side length of 12 mm and a serpentine wire design for the wire transmission line, thus realizing the tensile performance of the sensor. The electrode part of the sensor for sEMG acquisition is specially designed and is enlarged in Figure 1a for a detailed view. The electrodes—prepared from graphene—possess high sensitivity, good biocompatibility, and can monitor human physiological electrical signals for a long time. These properties make graphene an ideal material for electrodes for wearable medical devices. PDMS has been widely used in the field of flexible electronics’ research because of its good optical and chemical properties, simple processing, and low price; thus, PDMS was chosen as a flexible substrate material in this paper [60]. By using PDMS substrate and a laser-induced graphene and transfer process, we prepared a flexible single-lead graphene sEMG sensor; the specific fabrication process is shown in Figure 1b.

For the preparation process of flexible graphene sEMG sensors, the wafers were cleaned with isopropyl alcohol (IPA) and deionized water (DI water) and the residual isopropyl alcohol was blown off with a nitrogen gun, followed by heating the wafers at 110 °C for 5 min. After completing all patterns, PDMS (SYLGARD 184, Midland, MI, USA) base solution and curing agent was used to prepare liquid PDMS. For spin coating 10:1, liquid PDMS was spin coated on wafer A at 300 r/min for 3 s. Subsequently, wafer B was placed on a hot plate at 100 °C for 10 min to accelerate the solidification of PDMS. A 20:1 liquid PDMS was spin coated onto wafer B at 300 r/min and 800 r/min for 3 s and 5 s, respectively, followed by heating wafer B on a 100 °C hot plate for 10 min to accelerate PDMS solidification. The CO_2_ laser patterning of porous graphene on PI was conducted with a VLS2.30 universal laser system platform (Universal Cabling Systems Inc., Los Angeles, CA, USA): 10.6 µm wavelength of laser with a beam size of ≈120 µm, a pulse duration of ≈14 µs, a maximum power of 30 W, and a maximum scanning speed of ≈23 in·s^−1^. The laser system provides the options of controlling image density (DPI; number of lines per inch) from 1 to 7 and changing the PPI (pulse per inch) from 0 to 1000. In all the experiments, the setting was fixed at 10.5% of the maximum power and 11% of the maximum scanning speed. The image density was set as 6. The PPI was set as 1000. All the experiments were performed under ambient conditions. The PI film is laid flat on wafer A, with the PI layer facing upward and the LIG layer in contact with the PDMS. The excess is peeled off from the PDMS of wafer A at this point to complete the patterning process. A 20:1 liquid PDMS is spin coated onto wafer B containing the transferred pattern at 300 r/min and 800 r/min, with spin coating times of 3 s and 5 s. The final ultra-thin encapsulation layer is formed on top of the electrode layer. The PDMS of wafer B is heated and the encapsulation process is completed in 10 min, and the processed flexible sEMG sensor is uncovered from wafer B.

We completed the preparation of a flexible sEMG sensor based on graphene electrodes. Our paper is to achieve the prediction of muscle strength and active rehabilitation training control of a hand rehabilitation robot; hence, this requires a surface EMG signal acquisition system with good resistance to motion interference as well as with long-term monitoring stability. Toral et al. [14] have demonstrated that the LIG-based dry electrodes they developed, compared in the acquisition of ECG and EEG, show comparable performance to commercial electrodes, while they are more comfortable for the user and their manufacturing cost is lower. They can be used in cost-effective wearable devices to monitor biomedical variables. We therefore used a flexible sEMG sensor based on graphene electrodes to replace conventional AgCl electrodes to accomplish our acquisition of surface muscle electrical signals, prediction of muscle force magnitude, and active rehabilitation training control of hand rehabilitation robots. This sensor allows us to meet the acquisition performance with stability and accuracy, while being more comfortable and portable. In general, the ability to accurately and imperceptibly monitor surface electromyography signals is critical for medical professionals and patients.

### 2.2. Flexible sEMG Acquisition Hardware Preparation and Mechanical Property Testing

#### 2.2.1. Flexible sEMG Acquisition Hardware Preparation Process

**Figure 2:**
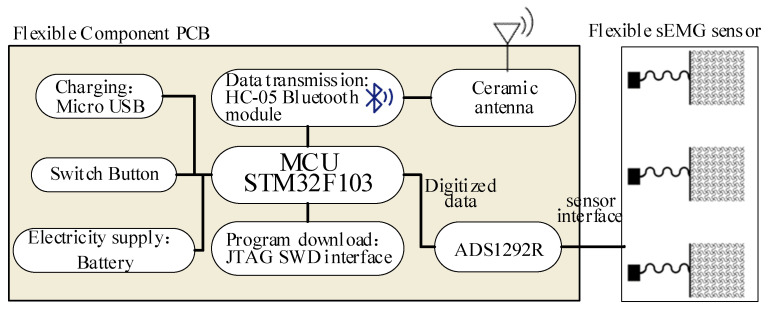
Overall framework figure of hardware.

The hardware of the sEMG acquisition system used in this paper uses Bluetooth communication, thus reducing the limitation of connection lines. The composition framework of the system hardware is shown in Figure 2, including circuit parts such as data acquisition, wireless transmission, and power management.

The signal processing circuit generally consists of a preamplifier circuit, a bandpass filter circuit, and an ADC acquisition circuit. The sEMG signal is weak due to its small amplitude and the increased impedance between the electrodes and the skin due to factors such as hair and skin tissue. Generally, the amplitude of the sEMG signal is 0–5 mV, so it cannot be directly converted to analog-to-digital. By using a preamplifier circuit, the sEMG signal is amplified and some interfering signals are filtered out. The most important filtering process is the working frequency of 50 Hz; removing it can improve the fidelity of the sEMG signal. Furthermore, since the effective sEMG signal is generally concentrated near the frequency of 150 Hz, a band-pass filter of 20–400 Hz can well avoid noise interference at high and low frequencies [61,62].

**Figure 3:**
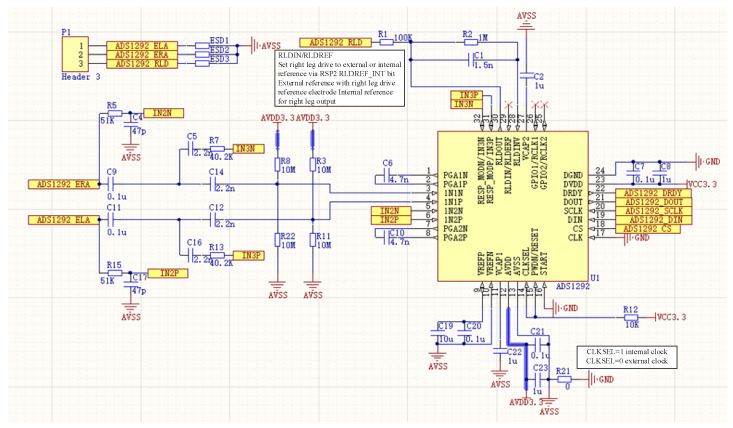
Schematic diagram of sEMG acquisition circuit based on ADS1292R.

The surface EMG signal frequency is 0–500 Hz, since most of our concern and useful EMG signal for muscle strength is within 250 Hz. The chosen low-cost low-power Bluetooth data transmission method is limited to 500 Hz for sampling frequency; therefore, TI’s ADS1292R is selected as the single-channel EMG acquisition amplifier chip, with sampling frequency set to 500 Hz, 24-bit analog-to-digital conversion, reference voltage of 2.42 V, system operating voltage of 3.4 V–4.2 V, and operating current of 45 mA during acquisition communication 3.7 V; its supporting front-end circuit principle is shown in Figure 3.

**Figure 4:**
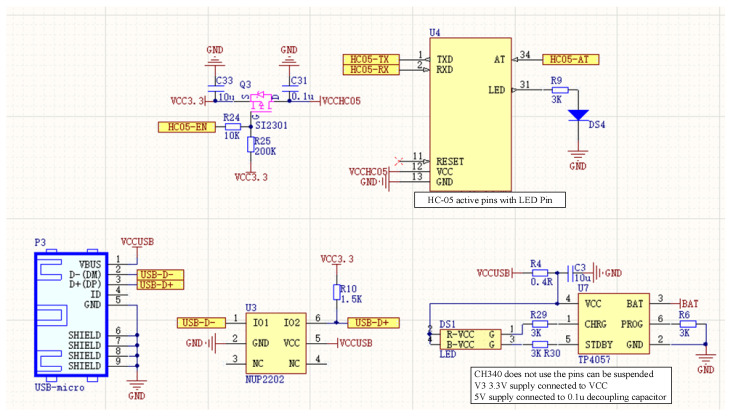
Bluetooth module and power circuit.

The signal transmission part uses a Bluetooth transmission method. Bluetooth is based on low-cost proximity wireless connection; low-power and micro-power consumption are its characteristics. The Bluetooth module used in this paper is an hc-05 Bluetooth module. The schematic diagram of the Bluetooth system is shown in Figure 4.

**Figure 5:**
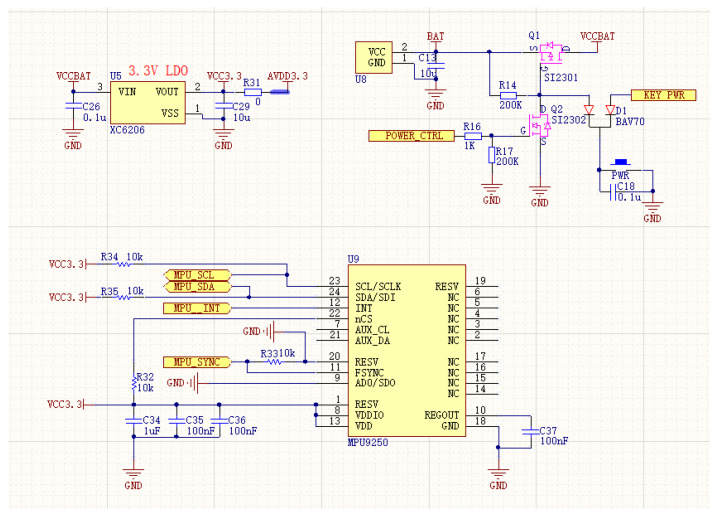
sEMG hardware using mpu9250 for motion monitoring.

At the same time, considering the subsequent use requirements, this paper adds a 9-axis acceleration sensor chip (model mpu9250, Tokyo, Japan) and the use schematic diagram is shown in Figure 5. In the follow-up research, it is planned to identify the large-scale movement of the hand by combining the acceleration sensing data in order to explore a wider application.

In order to realize the bendable performance of the hardware system, this paper realizes the circuit board through hardware design and flexible circuit technology. In this paper, sEMG hardware is divided into three areas. Each area is connected through wiring and the area without wiring is hollowed out.

**Figure 6:**
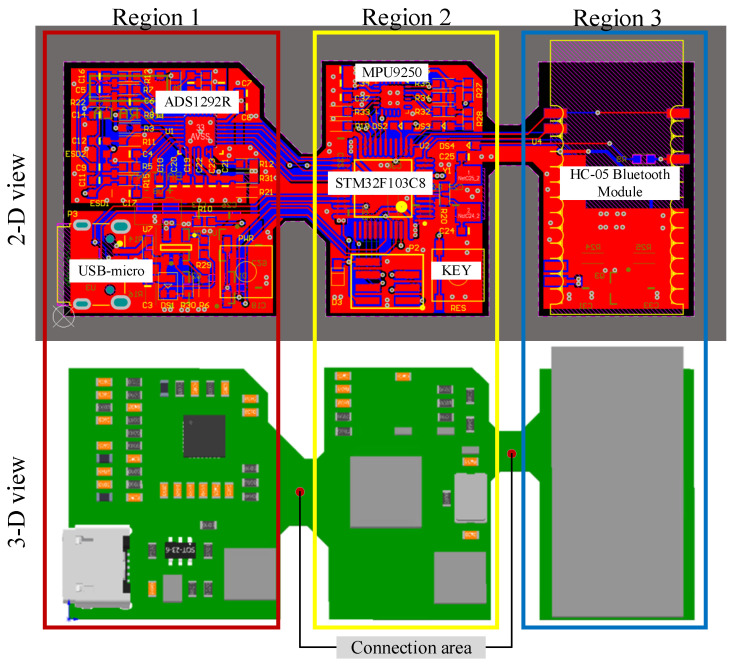
Circuit design drawing with dividing different areas.

The external network circuits for power management and charging lamps are placed in area 1. The main chip, indicators, and button interaction are placed in area 2. The Bluetooth module used in this paper is hc-05, which is independent of area 3 due to its size. The design diagram of the flexible partitioned circuit board is shown in Figure 6.

After completing the design of the sEMG acquisition circuit, the package is prepared using a flexible printed circuit board manufacturing process. A flexible printed circuit (FPC) has significant advantages as a special electronic interconnection technology. The main reason for the flexibility of FPC is the PI substrate supporting the copper foil and the copper foil with good bending properties.

**Figure 7:**
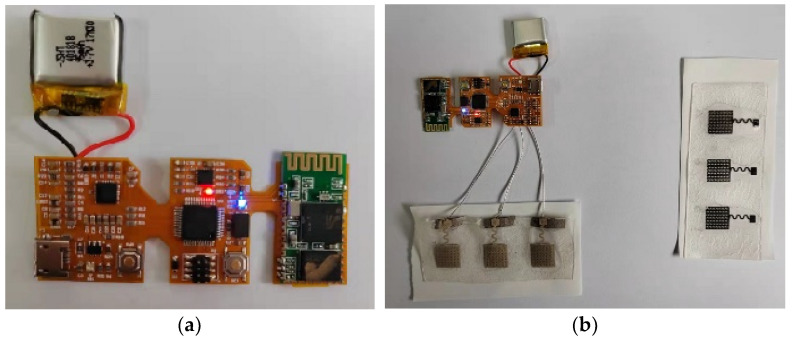
Flexible sEMG acquisition system. (**a**) Flexible circuit hardware; (**b**) hardware connected with single-lead sEMG sensor.

This paper completes the flexible sEMG acquisition hardware. The flexible lead can be connected with the flexible electromyography electrode designed in this paper. The whole flexible sEMG acquisition circuit is shown in Figure 7.

#### 2.2.2. Flexible sEMG Hardware Mechanical Performance Testing

Using the finite element software ABAQUS (Abaqus2016, SIMULIA, Velizy Villacoublay, France), we simulated the effects of these types of deformations during the stretching, bending, and twisting of the FPCB of the flexible sEMG hardware. First, we performed the experimental analysis of the flexible sEMG acquisition circuit board in an ABAQUS simulation environment for stretching, twisting, and bending. In the ABAQUS pre-processing, we will build the finite element model of the flexible printed circuit board according to the material properties of the copper layer and the polyimide (PI) layer of the required material for FPC [63]. The simulation is performed from both top and bottom directions, where the simulation parameters consist of the copper layer (density 8.9 g/cm³, modulus of elasticity 110 GPa, Poisson’s ratio 0.3) and the polyimide layer (density 1.15 g/cm³, modulus of elasticity 2673 MPa, Poisson’s ratio 0.36). Since the fixture is defined as a rigid body, there is no need to define material parameters in ABAQUS. The copper foil and the polyimide (PI) substrate are defined as elastic models. The constraint range set during the simulation was set roughly with reference to the curve of the human forearm acquisition position.

After setting up the pre-processing parameters, we then simulated the flexible sEMG acquisition board in an ABAQUS simulation environment for tensile, torsional, and bending experiments. In the ABAQUS post-processing, the results of the pre-processing of the flexible sEMG acquisition board are extracted and the stress value cloud of the FPCB is viewed to analyze the deformation trend and to summarize the distribution pattern, from which it is inferred whether the FPCB is damaged by the three deformation simulations.

### 2.3. Grip Force Prediction Algorithm and Hand Rehabilitation Control Method Based on Flexible sEMG Sensing System

**Figure 8:**
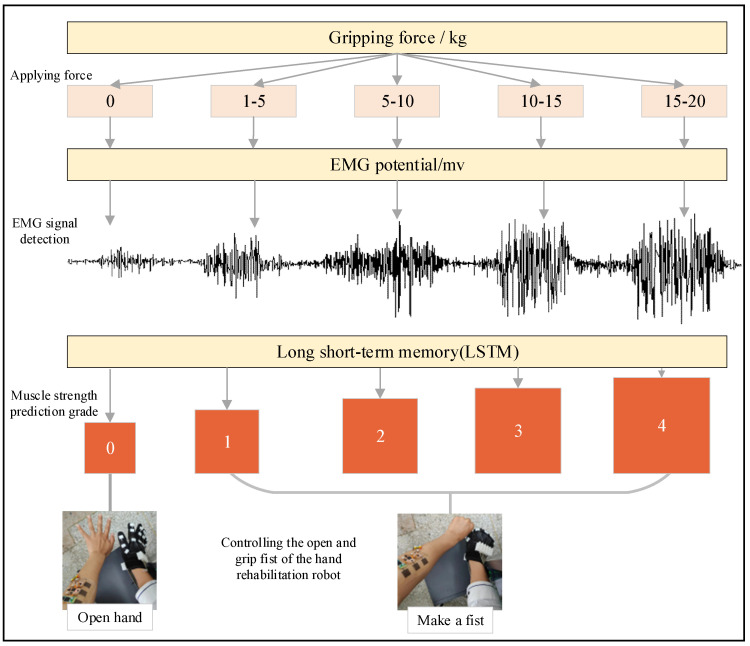
Flow chart of grip force prediction algorithm and hand rehabilitation control method.

Most current commercial force or torque sensors are bulky, expensive, and not user-friendly, nor do they make full use of the sensors for quantitative analysis of the relationship between lateral force and torque and electromyographic signals. The flexible sEMG acquisition system designed in this paper has the advantages of accurate data acquisition, high reliability, small size, low power consumption, simple operation, and no violation of wearing. In the control process of the hand function rehabilitation robot, two important features should be included, namely the movement of the motion and the strength magnitude of the motion. The current hand rehabilitation robots mostly control the robot after recognizing the motion pattern, ignoring the magnitude of the force applied to the hand, thus increasing the mental stress of the patient. The purpose of this part is to find a method to predict the hand strength magnitude based on EMG signals to provide a basis for the control of the hand rehabilitation force application magnitude. Figure 8 shows the flow chart of our proposed grip force prediction algorithm and hand rehabilitation control method based on a flexible sEMG sensing system. Among them, the grip force prediction algorithm is the most central part.

The processing of the sEMG signal is divided into time and frequency domains. Time domain analysis treats the EMG signal as a function of time. Statistical analysis is then performed with time as the independent variable, which can provide indicators to assess the characteristics of the change in the EMG curve in the time dimension. The time domain approach best reflects the root mean square (*RMS*) of the muscle strength information. Here, we used *RMS* as a characteristic of the EMG signal.

(1) $$RMS=\sqrt{\frac{1}{N}\sum_{i=1}^{N}d^{2}(i)}$$

In the equation, *d(i)* denotes the single channel sEMG signal sequence and *N* denotes the length of the sEMG signal sequence used to calculate the *RMS* value.

We use a long short-term memory network (LSTM) as a hand grasp strength prediction model. LSTM is a special kind of recurrent neural network (RNN), which is mainly designed to solve the gradient disappearance and gradient explosion problems during the training of long sequences. Simply speaking, it means that LSTM can have better performance in longer sequences compared with normal RNN. It is suitable for processing bioelectrical signals with time-series characteristics [64,65]. Further, it is also suitable for the time-series feature *RMS* we employ here. In the later sections, we will describe the model in detail. After the completion of the model for predicting the hand grip strength magnitude from the EMG signal, the obtained muscle strength levels are transmitted (via Bluetooth to serial transmission) to the main control chip of the hand rehabilitation device. Then, the force level is used as the threshold input to control the hand rehabilitation device to grip and open the hand. The muscle strength level 0 is the open position of the hand rehabilitation device. The muscle strength level 1 to 4 is the fist clenching position of the hand rehabilitation device, while the strength of the fist clenching gradually increases according to the rising level. The prototype hand rehabilitation robot appeared in our previous article and we also applied the designed flexible sEMG acquisition system to the training mode of the device to improve the hand rehabilitation effect [66].

### 2.4. Scenario Setting of Muscle Strength Prediction Experiment and sEMG Signal Processing Process

**Figure 9:**
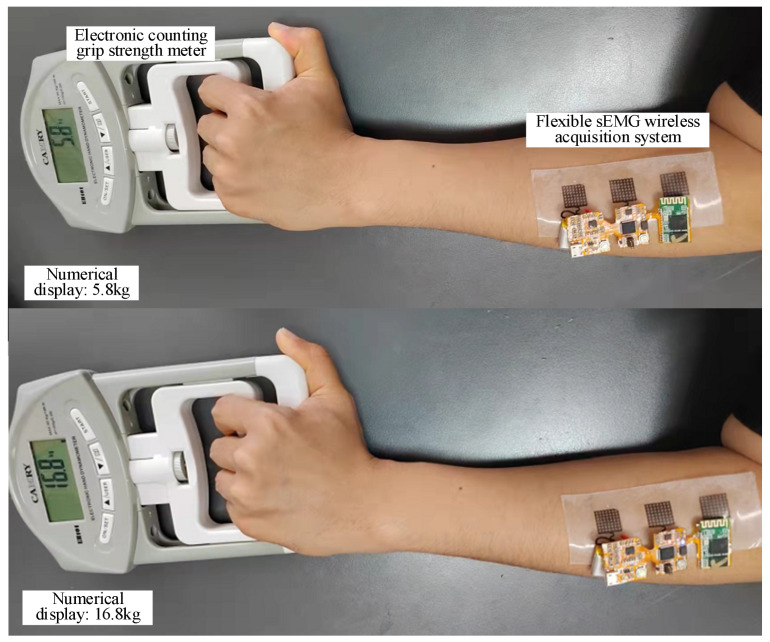
sEMG signals collected under different grip strength.

The purpose of this part is to find a method for predicting hand force magnitude based on EMG signals to provide a basis for the control of force magnitude applied by hand rehabilitation equipment. In order to verify the feasibility of the flexible sEMG acquisition system made in this project for detecting muscle force magnitude, we designed a grip strength testing and prediction experiment. The experiment was conducted in a laboratory setting, as shown in Figure 9. The subjects were two healthy males who had no previous history of neurological or muscular disorders. The subjects gave their consent to be included in the study prior to participating in this study. The experiment required the subjects to wear a flexible sEMG collection device developed in this study while holding an electronic counting grip in one hand for calibration testing. The calibration apparatus was a commercial electronic counting grip device TXUT-013 (CAMRY Company, Zhongshan, China), as shown in Figure 9, which has a range of 0–90 kg and has the function of real-time grip force display, acquisition, and storage. Since the grip gesture is mainly related to finger movements and its function is mainly controlled by the forearm muscle groups [28], the flexible sEMG collection device was placed centrally on the forearm with the subject’s forearm flat on the table. Prior to testing, 75% alcohol pads were gently rubbed on the skin of selected muscles to enhance conductivity [67].

**Figure 10:**
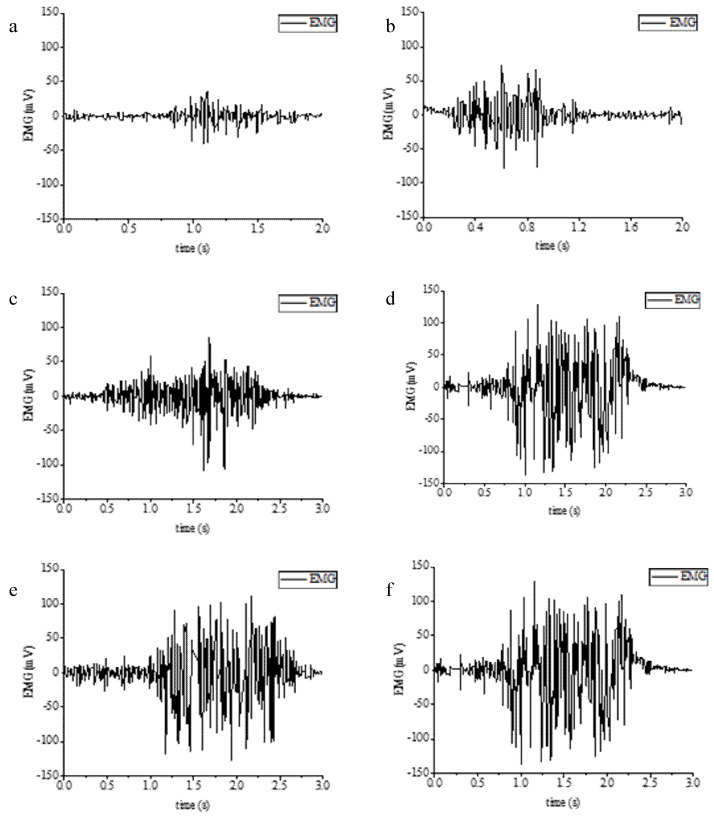
Electromyography under different grip strength. (**a**) Electromyography at 5 kg grip strength. (**b**) Electromyography at 10 kg. (**c**) Electromyography at 15 kg. (**d**) Electromyography at 20 kg. (**e**) Electromyography at 25 kg. (**f**) Electromyography at 30 kg.

After the experiment was prepared, the subjects grasped the force acquisition blocks according to six different levels of force (5 kg, 10 kg, 15 kg, 20 kg, 25 kg, and 30 kg) and the approximate constant force grip strength lasted for 20 s according to the value on the display of the electronic counting grip, with 30 sets of each applied force size. The typical sEMG waveforms at different grip strengths are shown in Figure 10. There are large differences in sEMG at different grip forces.

**Figure 11:**
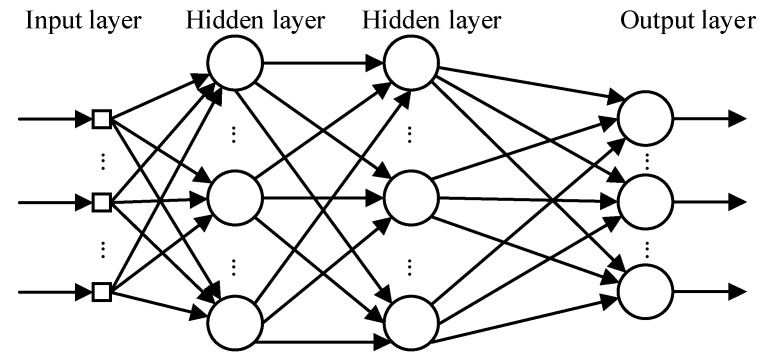
Neural network model.

Firstly, the collected data are cleaned and normalized in order to input the segmentation function. The purpose of the segmentation function is to divide the processed data and labels into a training set and a test set in a 4:1 ratio, where 80% is the training data set and 20% is the test set. The model learning rate is set to 0.02 and the cross entropy loss function is used. Then, the data are transformed into tensor, and the training set and test set data are encapsulated into train by dataloader_ Loader and test_ Loader to obtain data by batch, 200 pieces per batch. Train the training set data for 60 times, output the training loss value and training accuracy once every 200 batches, and save the weight parameter with the smallest loss value as best_ weights. Pt, the purpose is to save the minimum weight parameters of training in the training set, so as to directly take the trained model to test the model in the later stage. Figure 11 shows the neural network model.

**Figure 12:**
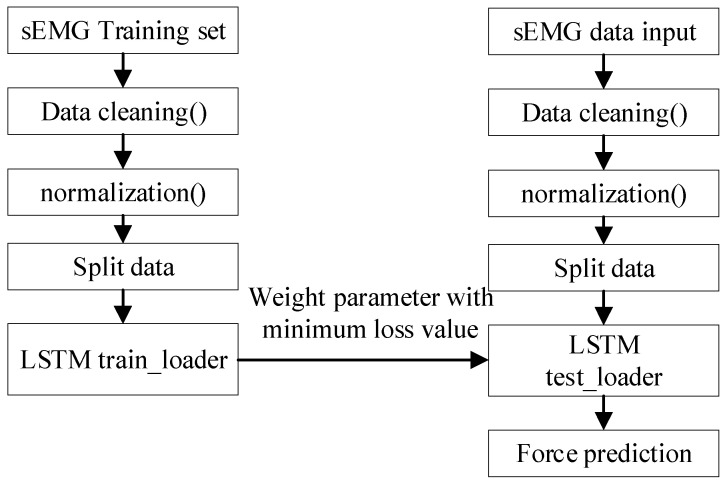
Force prediction using LSTM.

We use the long short-term memory (LSTM) algorithm; the algorithm flow is shown in Figure 12.

We classified muscle strength into four strength levels (0 kg, 5 kg, 10 kg, 15 kg, and 20 kg) and predicted which level the measurements were close to. It is also compared with the effectiveness of back propagation network (BP-ANN)—an algorithm widely used to train feed-forward neural networks for supervised learning—effectively calculating the gradient of the loss function with respect to the network weights. This makes it possible to train multilayer networks using a gradient approach to update weights to minimize losses. The gradient of the loss function with respect to each weight is computed by a chain rule that iterates backward one at a time starting from the last layer so that the error function decreases in the direction of the negative gradient, thus allowing the BP neural network prediction output to continuously approximate the desired output [68].

## 3. Results

### 3.1. Flexible sEMG Hardware Mechanical Performance Test Results

**Figure 13:**
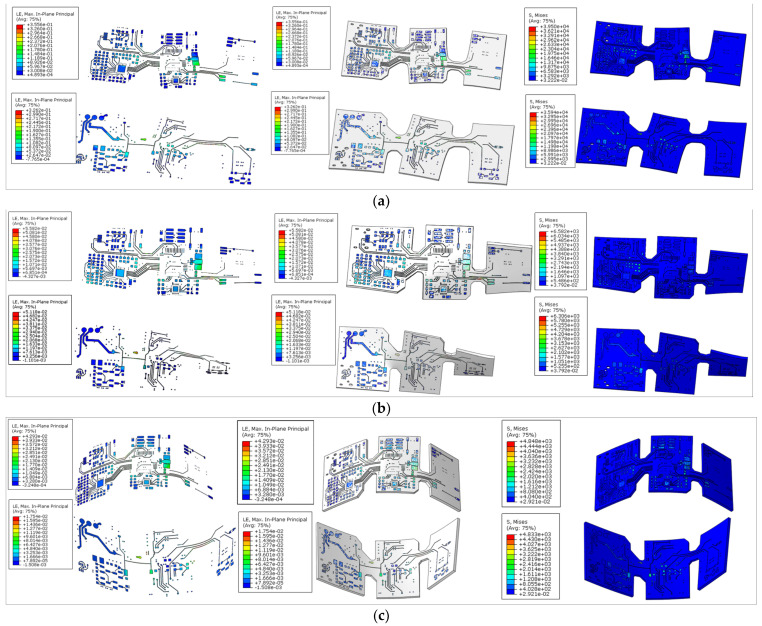
Hardware simulation diagram. (**a**) Tensile test: constrain one end and stretch the other end by 8 mm. (**b**) Torsion test: constrain one end and rotate the other end by 90 degrees (1.57 radians). (**c**) Bending test: bending jig restraint, both ends downward displacement of 15 mm.

The experiment of the stress value cloud, as shown in Figure 13, indicates where the stress increases with the increase in board strain. It is obvious that the FPCB still maintains good tensile and bending and torsional properties after three kinds of deformation simulation.

### 3.2. Overall Display of Flexible sEMG Collection System

**Figure 14:**
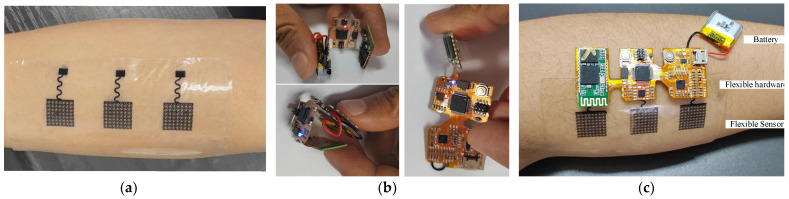
(**a**) The flexible sEMG sensor based on PMDS has good adhesion. (**b**) The hardware has very good bending and twisting characteristics. (**c**) Prototype of wireless sEMG acquisition system.

In this section, we first show a practical scenario of flexible sEMG sensing attached to a human forearm, where the sensor has good adhesion due to intermolecular forces (van der Waals forces) [69], as shown in Figure 14a. Then, we show, through Figure 14b, the actual scene of bending and twisting the flexible sEMG acquisition circuit board by two fingers. Finally, we show in Figure 14c the actual scene of the whole flexible sEMG acquisition system affixed to the human forearm. We combine the mechanical performance simulation experiments of the flexible sEMG sensor and acquisition hardware in the previous section with the real application scenario demonstration in this section. The combination of simulation and real application scenarios can further determine that the flexible stretchable sEMG acquisition system has good tensile, torsional, and bending properties.

### 3.3. Results of Grip Force Prediction Algorithm and Hand Rehabilitation Control Method Based on Flexible sEMG Acquisition System

**Figure 15:**
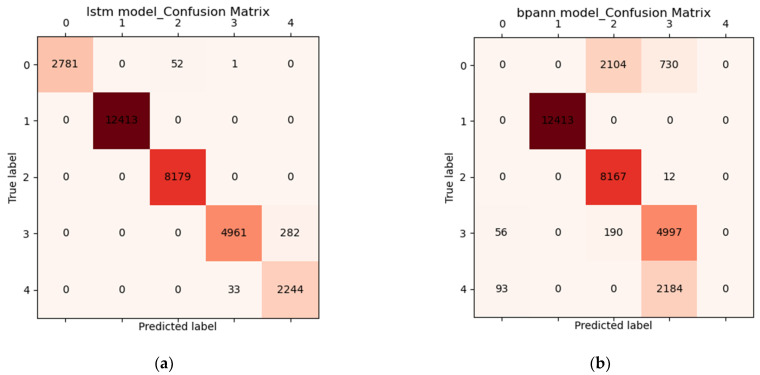
Confusion matrix for different types of prediction models. (**a**) Classification prediction model based on LSTM algorithm. (**b**) Classification prediction model based on BP-ANN algorithm.

We tested two individuals, and a total of 30,946 offline data were validated. Offline validation of the proposed algorithm using back propagation network (BP-ANN) determinations achieved an accuracy of 82.65%. When we used the long short-term memory (LSTM) algorithm, we obtained an accuracy of 98.81%. Figure 15 shows the confusion matrix results for the two prediction models, LSTM and BP-ANN. We used python to directly plot the confusion matrix after we obtained the test results, as shown in Figure 15.

**Figure 16:**
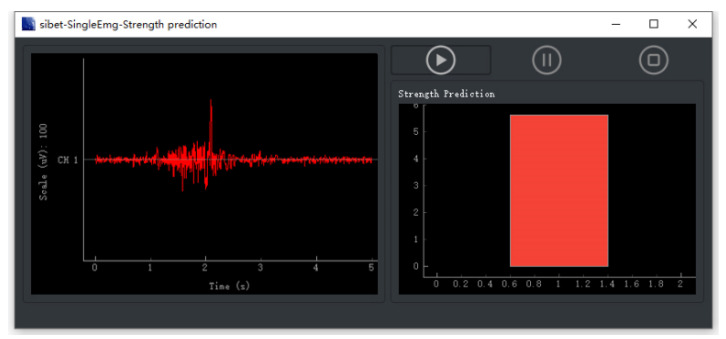
The strength prediction UI use single sEMG system.

We use PyQt5 as the framework of the software interface to build a simple sEMG ac-quisition and display software. In the process, the interface will store and process the original data in real time. The single-lead sEMG acquisition software is shown in Figure 16.

**Figure 17:**
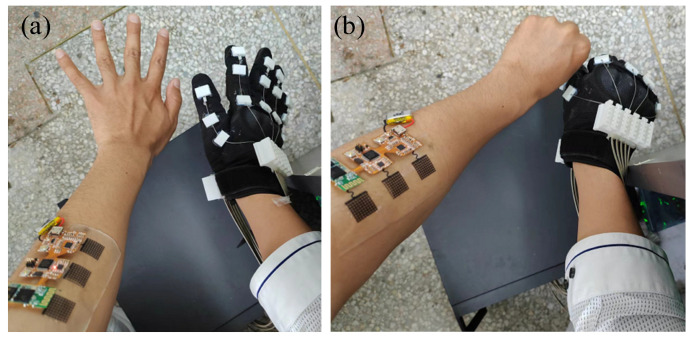
Rehabilitation application using flexible sEMG system. (**a**) Myoelectric signals from the left forearm control the opening of the right hand rehabilitation robot. (**b**) Myoelectric signals from the left forearm control the right hand rehabilitation robot to make a fist.

For the needs of mirror training in general rehabilitation training, this paper uses a flexible sEMG acquisition system to recognize two simple gestures, including grasping and stretching. The flexible sEMG acquisition system is used to collect the sEMG signal of one hand, to judge whether the hand is grasped or extended, and to control the other hand through the exoskeleton hand rehabilitation robot, as shown in Figure 17.

## 4. Discussion

The flexible and stretchable sEMG sensor and sensing system designed in this paper has certain flexibility and the system shows good bending and twisting characteristics. By using the flexible electronic system shown in this paper, the hand muscle strength in rehabilitation training or daily life can be monitored. At the same time, because the sEMG system designed in this paper has good electrical performance, it can better use the flexible sEMG system designed in this paper for rehabilitation training or gesture recognition.

However, the system in this paper is still in the principle prototype and there is still a certain distance from a fully mature product. The designed hardware shows good bending and twisting characteristics but lacks extensibility. In the follow-up, we can further develop the flexible circuit board. By optimizing the routing design, the lead wire between the three-lead hardware is designed as a serpentine wire in order to realize the tensile characteristics of the whole system.

In the follow-up research, we hope to make the flexible sEMG sensor into an integral column sensor and to expand the hardware from a single-lead sEMG to array a multi-lead sEMG system. Through arraying sEMG perception, more muscle-related parameters can be obtained, which will achieve higher accuracy and more motion prediction.

## 5. Conclusions

In this paper, we developed a flexible sEMG sensor for muscle strength evaluation and rehabilitation training. At the same time, we developed flexible wireless sEMG acquisition hardware to realize the flexibility of the overall sEMG acquisition system. Through the fully flexible acquisition system, the collected signal has good stability. Through the extraction of sEMG characteristics under different muscle strengths, the project realizes the research of muscle strength feedback through sEMG and realizes the mirror rehabilitation.

## Data Availability

The data presented in this study are available on request from the corresponding author.
